# Strategies for improved isopropanol–butanol production by a *Clostridium* strain from glucose and hemicellulose through consolidated bioprocessing

**DOI:** 10.1186/s13068-017-0805-1

**Published:** 2017-05-08

**Authors:** Fengxue Xin, Tianpeng Chen, Yujiang Jiang, Weiliang Dong, Wenming Zhang, Min Zhang, Hao Wu, Jiangfeng Ma, Min Jiang

**Affiliations:** 1 State Key Laboratory of Materials-Oriented Chemical Engineering, College of Biotechnology and Pharmaceutical Engineering, Nanjing Tech University, Puzhu South Road 30#, 211816 Nanjing, People’s Republic of China; 2 Jiangsu National Synergetic Innovation Center for Advanced Materials (SICAM), Nanjing Tech University, 211816 Nanjing, People’s Republic of China

**Keywords:** *Clostridium* sp., Butanol, Isopropanol, In situ extraction, Polysaccharides, Temperature shift, Xylanase, Consolidated bioprocessing

## Abstract

**Background:**

High cost of traditional substrates and formation of by-products (such as acetone and ethanol) in acetone–butanol–ethanol (ABE) fermentation hindered the large-scale production of biobutanol. Here, we comprehensively characterized a newly isolated solventogenic and xylanolytic *Clostridium* species, which could produce butanol at a high ratio with elimination of ethanol and conversion of acetone to more value-added product, isopropanol. Ultimately, direct butanol production from hemicellulose was achieved with efficient expression of indigenous xylanase by the novel strain via consolidated bioprocessing.

**Results:**

A novel wild-type *Clostridium* sp. strain NJP7 was isolated and characterized in this study, which was capable of fermenting monosaccharides, e.g., glucose into butanol via a fermentative acetone–isopropanol–butanol pathway. With enhancement of buffering capacity and alcohol dehydrogenase activities, butanol and isopropanol titer by *Clostridium* sp. strain NJP7 was improved to 12.21 and 1.92 g/L, respectively, and solvent productivity could be enhanced to 0.44 g/L/h. Furthermore, with in situ extraction with biodiesel, the amount of butanol and isopropanol was finally improved to 25.58 and 5.25 g/L in the fed-batch mode. Meanwhile, *Clostridium* sp. strain NJP7 shows capability of direct isopropanol–butanol production from hemicelluloses with expression of indigenous xylanase. 2.06 g/L of butanol and 0.54 g/L of isopropanol were finally achieved through the temperature-shift simultaneous saccharification and fermentation, representing the highest butanol production directly from hemicellulose.

**Conclusion:**

The co-production of isopropanol with butanol by the newly isolated *Clostridium* sp. strain NJP7 would add on the economical values for butanol fermentation. Furthermore, the high isopropanol-butanol production with in situ extraction would also greatly enhance the economic feasibility for fermentative production of butanol–isopropanol in large scale. Meanwhile, its direct production of butanol–isopropanol from polysaccharides, hemicellulose through secretion of indigenous thermostable xylanase, shows great potential using lignocellulosic wastes for biofuel production.

## Background

As an important industrial chemical, butanol has been gaining increased attention as an alternative fuel due to its higher energy density, less hygroscopy, better compatibility with current car engine, etc. Butanol can be produced anaerobically by several solventogenic bacteria belonging to the genus of *Clostridium*. Currently, the most extensively studied species are *C. acetobutylicum* and *C. beijerinckii*, which produce butanol, acetone, and ethanol with a typical ratio of 6:3:1 in a so-called acetone–butanol–ethanol (ABE) fermentation process [1, 2]. Although solventogenic *Clostridium* species have been used for large-scale ABE fermentation, butanol production is still considered less economical than ethanol fermentation using yeasts. Since acetone cannot be used as a fuel due to its corrosiveness to car engine parts that are composed of rubber or plastic, its co-production with butanol (and ethanol) is viewed as undesirable because it reduces the butanol yield [3]. Accordingly, much effort has focused on reduction or elimination of acetone by interrupting the acetone pathway. However, disruption of the genes responsible for acetone formation led not only to less acetone production, but also decreased butanol production with the accumulation of volatile acids (e.g., acetate and butyrate) [2–4].

Recently, an alternative strategy to improve the economic efficacy of butanol production is to convert acetone to more value-added product, such as isopropanol through introduction of an isopropanol-synthesizing gene, which possesses a higher energy density than acetone (23.9 vs 22.6 MJ/L) and shows broader usage as fuel, solvents, and chemical intermediates [4, 5]. In this process, a NADPH-dependent secondary alcohol dehydrogenase (s-ADH) was expressed in ABE-generating *Clostridium* and efficiently converted acetone into isopropanol, hence switching ABE fermentation into isopropanol–butanol–ethanol (IBE) fermentation [4, 6]. Actually, in nature, several *C. beijerinckii* strains, such as *C. beijerinckii* NRRL B-593, were shown to indigenously produce isopropanol without acetone formation, but the titer ranges of isopropanol and butanol are not conclusive based on different studies [7]. According to Survase et al., *C. beijerinckii* NRRL B-593 produced 2.2 and 3.7 g/L of isopropanol and butanol, respectively, in the batch culture containing 60 g/L of initial glucose [8]. Shaheen et al. demonstrated that *C. beijerinckii* NRRL B-592 produced about 16 g/L of total solvents including isopropanol from 80 g/L of maize mash, but the efficiency of acetone conversion is still unknown [9]. Recently, Ng et al. reported ethanol–butanol–isopropanol production in *C. beijerinckii* NRRL B-592 from 60 g/L of glucose with a final titer of 1.85, 8.13, and 2.22 g/L, respectively [10]. Conversely, strain NRRL B-592 did not show any isopropanol production in another study [7]. So comprehensive investigations are still needed to further elaborate biosolvent production profiles for wild-type isopropanol–butanol-producing *Clostridium*.

Another bottleneck hindering scaling up of butanol production is the cost of the traditional feedstocks [3, 11]. Although solventogenic *Clostridium* sp. utilizes a broad range of monosaccharides, disaccharides, starches, and other substrates, such as inulin, pectin, and whey, they cannot directly utilize cellulose and hemicellulose for butanol production [2]. With the catalysis of lignocellulose-degrading enzymes, such as cellulase and xylanase, solventogenic *Clostridium* sp. could ferment the component sugars (hexoses and pentoses) in the lignocellulosic hydrolysate to biobutanol [12]. However, the high costs of lignocellulose-degrading enzymes would increase the overall economics [13, 14]. Producing biobutanol directly from cellulose or hemicelluloses without addition of exogenous cellulase/xylanase, known as consolidated bioprocessing (CBP), is believed to reduce costs substantially compared to a process in which cellulose/hemicellulose degradation and fermentation to butanol are accomplished in separate steps. Therefore, novel bacterial isolation with both solventogenic and cellulose/hemicellulose-degrading properties is still needed to achieve direct butanol production from lignobiomass.

Here, we first reported a newly isolated hemicellulose-degrading and solventogenic *Clostridium* sp. strain NJP7. Unlike other reported solventogenic *Clostridium*, this strain could synthesize butanol via a fermentative acetone–isopropanol–butanol (AIB) pathway. More importantly, strain NJP7 could directly achieve butanol production from hemicellulose in CBP. It was also attempted to further increase the final butanol and isopropanol titer and productivity by employing exogenous acids and reducing factors and in situ extraction with biodiesel. Lastly, hemicellulose-degrading enzymes—xylanases from strain NJP7 were characterized and a further temperature-shift strategy was designed to further increase butanol production from hemicelluloses.

## Results and discussion

### Characterization of novel hemicellulose-degrading and isopropanol–butanol-generating *Clostridium* sp. strain NJP7

**Fig. 1:**
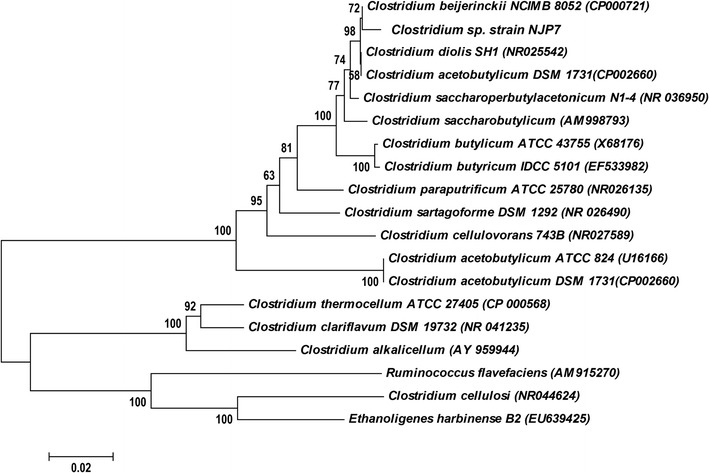
Phylogenetic tree of *Clostridium* sp. strain NJP7 using the neighbor-joining method (MEGA 4.0) based on its 16S rRNA gene sequences

In order to obtain hemicellulose-degrading and butanol-generating microbe, microbial communities were set up with inocula from decompose soil. After anaerobic incubation of 24 h in serum bottles fed with 10 g/L xylan, serial dilution of the culture medium was carried out. The diluted supernatant of 0.1 mL was then spread onto agar plates amended with 10 g/L of xylan and incubated for 48 h anaerobically to obtain individual colonies. Since xylanase activities could be determined by the size of orange digestion halos formed on xylan plates using Congo red staining, violet colonies were picked and inoculated individually to reduced mineral medium spiked with 30 g/L of glucose for butanol production. Among the cultures, nine colonies with big orange digestion halos were selected, and then conducted three more transfers on agar plates to ensure their purities. The capability of butanol generation was tested under the batch fermentation mode fed with 30 g/L of glucose. During the nine colonies, only one (strain NJP7) showed butanol production. By detecting with GC-FID and HPLC, the main metabolic products were identified as acetone, isopropanol, butanol, acetic acid, and butyric acid, indicating that strain NJP7 synthesizes biobutanol via a fermentative acetone–isopropanol–butanol (AIB) pathway. Strain NJP7 also represents the first wild-type xylanolytic and isopropanol–butanol producing bacterium, which would potentially provide some genetic or enzymatic tools, such as isopropanol-generating genes or xylanases. Further phylogenetic analysis of the 16S rRNA genes showed that this isolate shares 99% sequence identity with the 16S rRNA gene of *C. beijerinckii* NCIMB 8052 (Fig. 1) and designated as *Clostridium* sp. strain NJP7. Nevertheless, strain NJP7 shows distinguished difference from *C. beijerinckii* NCIMB 8052 by its physiological differences in metabolite production, whose metabolic products are mainly composed of acetone, butanol, and ethanol.

### Dual functions of exogenous acids on improvement of butanol production by *Clostridium* sp. strain NJP7

**Fig. 2:**
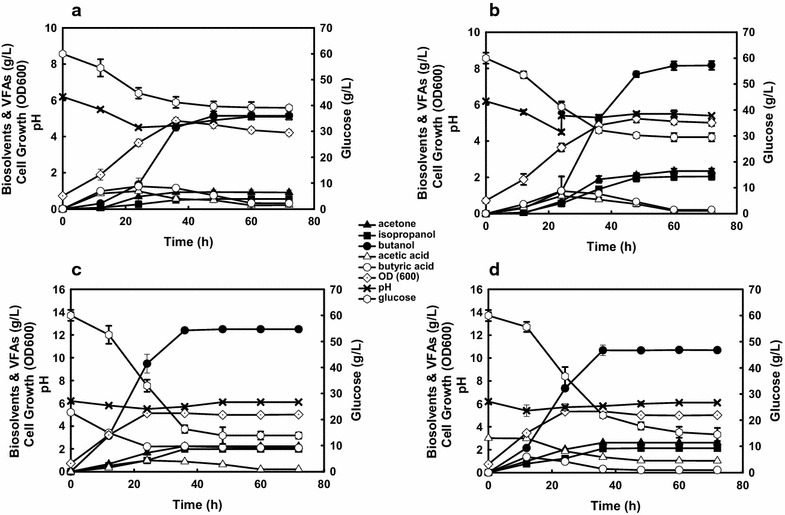
Growth and fermentation profiles of *Clostridium* sp. strain NJP7 in reduced mineral medium containing 60 g/L of glucose without pH adjustment (**a**), with pH adjustment (**b**), with addition of 60 mM of butyrate (**c**), and with addition of 50 mM of acetate (**d**)

To investigate the butanol production potential by *Clostridium* sp. strain NJP7, batch experiments in triplicates were firstly conducted in mineral salts medium spiked with 60 g/L of glucose without pH adjustment. As shown in Fig. 2a, a classical butanol fermentation pattern was observed, with a first stage of acids production accompanying with the decrease of pH values (6.2–4.5) and fast cell growth (OD600 = 4.5) during the first 24 h, followed by a second stage of solvent formation with reutilization of part of the acids, as also reflected by the renounce of pH values (4.5–5.3). After 48 h of fermentation, strain NJP7 could produce 0.93 g/L of acetone, 0.55 g/L of isopropanol, and 5.14 g/L of butanol, with negligible amount of ethanol. 20.56 g/L of glucose was consumed with butanol yield of 0.25 g/g. The ratio of acetone:isopropanol:butanol is 1.7:1:9 with 77% of butanol, which is much higher as compared to that (60%) in the typical ABE fermentation, indicating that strain NJP7 would show great potential in large-scale butanol production with increased butanol yield. However, further attempts are still needed to improve butanol production and productivity.

The efficiency of the bioconversion of substrates into butanol with solventogenic *Clostridia* could be increased by adjusting the metabolism. Exogenous acids (e.g., acetate and butyrate) have been reported to affect butanol production. However, their effects on butanol production were still inconclusive based on different studies. Taking *C. beijerinckii* NCIMB 8052 as an example, *Chen and Blaschek* found that the addition of acetate (20–80 mM) could increase solvent production and prevent strain degeneration [15]. However, Holt et al. reported that the addition of 20 mM acetate caused a decrease in butanol concentration by half [16]. Although the addition of 10 mM butyrate was found to initiate solvent production during the early exponential growth phase, there was no increase in the final solvent concentration [16]. So in order to further improve butanol production by *Clostridium* sp. strain NJP7 and elaborate the effects of exogenous acids on solvent production, different amounts of sodium butyric and acetic (20–70 mM) were added into the mineral salts medium containing 60 g/L of glucose, respectively (Table 1). Interestingly, the results showed that both butyrate and acetate could obviously improve butanol production and AIB yield for culture NJP7, and the final butanol titer and AIB yield could be significantly enhanced to 11.07 and 10.71 g/L and 0.37 and 0.36 g/g as compared to the control batch (5.14 g/L and 0.33 g/g), when 60 mM of butyrate or 50 mM of acetate was supplemented, respectively. However, further increase of exogenous acids did not show butanol improvement. When butyrate or acetate was introduced into the medium, the supplemented exogenous acids were rapidly re-assimilated, leading to an increased butanol production during the early exponential phase (24 h) as compared to the control. For instance, when 60 mM of butyrate was added into the medium, strain NJP7 could produce 9.11 g/L of butanol during the first 24 h, which is much higher as compared to 1.23 g/L of the control (Fig. 2a, c). Subsequently, a 49.01% increase of enzymatic activity was also observed for NADH-dependent butanol dehydrogenase (BDH), implying that the added butyrate could more efficiently induce the genetic expression of butanol-forming enzymes and hence initiate butanol production. Similar results were also obtained when acetate was added into the medium as shown in Table 1. The addition of 50 mM of acetate could not only accelerate the final butanol production (10.71 vs 5.14 g/L) and glucose consumption (20.18 vs 42.89 g/L) when compared to the control culture, but also improve the BDH activities (0.49 vs 0.26 U/mg), triggering the metabolic shift from acetogenesis to solventogenesis earlier (12 vs 24 h) (Fig. 2d). Meanwhile, the ratios of acetone:isopropanol:butanol were also influenced accordingly. With increase of butyrate to 60 mM, the approximate ratio of acetone:isopropanol was still 1.6:1. However, the addition of acetate would favor the increase of acetone ratio instead of butanol, suggesting that the additional acetate might also shift the metabolic pathway toward acetone production. This was consistent with previous report that the addition of acetate enhanced CoA-transferase activity within *C. beijerinckii* NCIMB 8052, thus leading to more acetone production [15].

**Table 1:** Effect of different amounts of sodium butyric and acetic on AIB production by wild-type *Clostridium* sp. strain NJP7

Fermentation parameters	Sodium butyric (mM)						Sodium acetic (mM)				
	Control	20	40	50	60	70	20	40	50	60	70
Acetone (g/L)	0.93	1.18	1.91	2.32	2.83	2.76	2.03	2.41	2.61	2.53	2.59
Isopropanol (g/L)	0.55	0.86	1.23	1.66	1.78	1.75	1.93	2.04	2.12	1.98	2.06
Butanol (g/L)	5.14	6.78	8.95	10.35	11.07	11.03	5.98	8.23	10.71	10.68	9.70
Acetate (g/L)	0.21	0.22	0.18	0.21	0.18	0.25	0.78	0.98	1.15	1.02	1.98
Butyrate (g/L)	0.41	1.12	2.06	2.12	2.15	3.56	0.35	0.37	0.21	0.31	0.21
AIB productivity (g/L/h)	0.14^a^	0.18^a^	0.34^b^	0.40^b^	0.44^b^	0.43^b^	0.21^a^	0.26^b^	0.40^b^	0.42^b^	0.40^b^
Glucose consumption (g/L)	20.18	25.94	35.56	39.81	42.35	42.03	30.12	39.29	42.89	42.19	41.07
AIB yield (g/g)	0.33	0.34	0.34	0.36	0.37	0.37	0.33	0.34	0.36	0.36	0.35
pH^c^	4.5	4.7	5.0	5.4	5.6	5.7	4.6	5.1	5.4	5.6	5.7
Final pH	5.3	5.6	5.8	6.0	6.1	6.1	5.3	5.5	5.9	6.1	6.2
ADH^d^ (U/mg)	0.26	0.31	0.39	0.45	0.51	0.51	0.27	0.37	0.49	0.48	0.47

*Control* fermentation without addition of any exogenous acids and pH adjustment

*Other experiments* different amounts of acetate or butyrate without pH adjustment were added

^a^Fermentation was complete at 48 h

^b^Fermentation was complete at 36 h

^c^The lowest pH values during the fermentation process

^d^Enzymatic activities at exponential phase

Unlike the enhanced butanol production with 60 mM butyrate or 50 mM acetate, only slightly improved butanol production was observed when 20 mM of butyrate or acetate was added into the medium. It should be noteworthy that the higher the amounts of butyrate or acetate supplemented, the higher the lowest pH values occurred during the fermentation process (Table 1). For example, the lowest pH values were 4.5, 4.7, 5.2, 5.5, 5.6, and 5.6 when butyrate concentrations increased from 0 to 70 mM. It is known that pH has been recognized as a key factor in determining the final titer of ABE fermentation, and lower pH (4.0–4.5) will cause a loss in the viability before the culture has a chance to switch to the solventogenesis phase. Compared to the control batch without pH adjustment, when pH values were adjusted back to 5.5 after 24 h of fermentation, the final butanol production could be further improved to 8.11 g/L even without supplementation of exogenous acids (Fig. 2b). On the contrary, with supplementation of 60 mM butyrate or 50 mM acetate, the pH values were always greater than 5.5 during the fermentation in spite of the acid production due to the buffering capacity of the acetate or butyrate added. Under such conditions, strain NJP7 could successfully switch to solventogenesis earlier, which shortened the fermentation duration to 36 h as compared to 48 h in the control. Hence, the AIB productivity could be further increased to 0.44 and 0.42 g/L/h with supplementation of 60 mM butyrate or 50 mM acetate, respectively, which is 114 and 100% higher than the control (0.14 g/L/h). Based on these results, it seems likely that the enhanced butanol production is due to both the increase of the buffering capacity as well as the shift in the metabolic pathways toward butanol production when butyrate or acetate was added to the medium.

### Manipulation of metabolic flux for further improvement of isopropanol–butanol production by *Clostridium* sp. strain NJP7

**Fig. 3:**
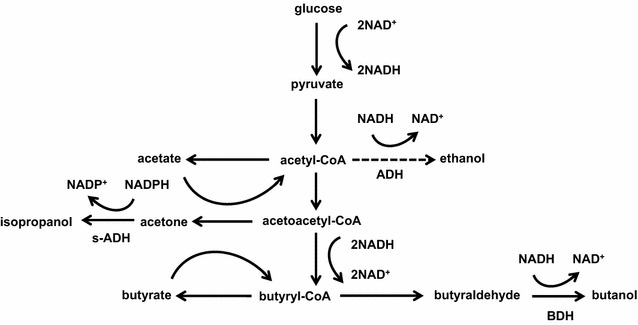
Proposed metabolic pathway for acetone–isopropanol–butanol (AIB) production within *Clostridium* sp. strain NJP7. *Dotted arrows* indicate reactions when using polysaccharides, such as hemicelluloses as the substrate

Isopropanol was synthesized by a NADPH-dependent s-ADH, which could catalyze acetone to isopropanol in some wild-type isopropanol-generating *Clostridium* species [4, 6]. Currently, the most extensively characterized s-ADH was from *C. beijerinckii* NRRL B-593, which was commonly introduced into *C. acetobutylicum* for conversion of acetone to isopropanol [7, 17]. The existence of isopropanol in the final metabolic product of *Clostridium* sp. strain NJP7 suggests that it also possesses a s-ADH, which will provide another alternative gene tool for other studies (Fig. 3). Unlike isopropanol production, butanol formation was conducted by NADH-dependent butanol BDH. Consequently, a lack or inefficient regeneration of cofactors of NADPH and NADH would shut down s-ADH and BDH activities. So in order to further increase butanol and isopropanol titers, it is crucial to increase NADPH and NADH pools to achieve a redox balance.

Vitamin B3 (VB3), also called nicotinamide is the precursor of both NAD and NADP, whose supplementation in the defined medium will increase the NADPH and NADH pool size. Accordingly, its effects on NAD-dependent s-ADH and NADPH-dependent BDH activities and changes in metabolites were assessed. As shown in Table 2, supplementation of VB3 could obviously improve s-ADH and BDH activities and solvent (isopropanol and butanol) production. Meanwhile, the ratio of isopropanol:acetone was also changed. NADPH-dependent s-ADH activities within strain NJP7 were 1.5 times higher in the presence of 20 mg/L of VB3, leading to the increase of isopropanol production (0.72 vs 0.55 g/L) and higher ratios of isopropanol:acetone (0.81:1 vs 0.59:1) than those in the control. Meanwhile, NADH-dependent BDH activities were also improved significantly. 1.6 times increase of BDH was observed in the presence of VB3 than that in the control and the final butanol production of 6.28 g/L occurred in the medium broth. The results demonstrated that the availabilities of reducing cofactors generated from VB3 could determine the amount of isopropanol and butanol production by wild-type *Clostridium* sp. strain NJP7. When both exogenous butyrate and VB3 were added into the medium, all parameters for metabolic products, enzymatic activities, and cell growth increased significantly. Cell growth was enhanced drastically to 5.12 of OD (A600), and corresponding BDH activities achieved 0.58 U/mg. These contribute to 12.21 g/L of butanol, 2.21 g/L of acetone, and 1.92 g/L of isopropanol. On the other hand, a 12% increase of s-ADH further improve the ratio of isopropanol:acetone, and finally reached 0.87:1. Although this AIB fermentation process conducted by strain NJP7 with supplementation of chemically synthesized butyrate and VB3 would have less profits compared to the one without addition of any ones, we , however, believed that the decreased gross profits could be compensated by the high AIB productivity, butanol ratio, and production of isopropanol. Furthermore, the costs caused by supplementation of butyrate could be further reduced by the addition of butyrate fermentative supernatant or co-culturing with other butyrate-generating bacterium.

**Table 2:** Metabolic products and enzymatic activities of *Clostridium* sp. strain NJP7

	Control	With VB3	With butyrate + VB3
Acetone (g/L)	0.93	0.89	2.21
Isopropanol (g/L)	0.55	0.72	1.92
Butanol (g/L)	5.14	6.28	12.21
Ratio of isopropanol:acetone	0.59:1	0.81:1	0.87:1
Glucose consumption (g/L)	20.18	21.87	41.98
AIB yield (g/g)	0.33	0.36	0.39
Max. OD (A600)	4.51	4.98	5.62
s-ADH^a^ (U/mg)	0.19	0.31	0.46
ADH^a^ (U/mg)	0.26	0.32	0.58

*Control* fermentation without addition of any exogenous acids and pH adjustment

*Other experiments* fermentation with addition of VB3 (20 mg/L) or butyrate (60 mM) and VB3(20 mg/L) without pH adjustment

^a^All of the samples were collected at their exponential phases for detection of their s-ADH and ADH activities: 24 h for Control and With VB3; 18 h for W With butyrate and VB3

### High isopropanol–butanol production with in situ extraction using biodiesel

**Fig. 4:**
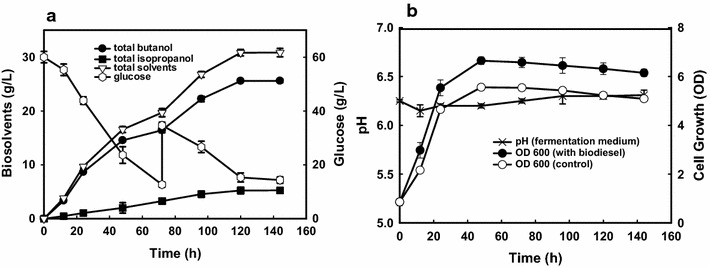
**a** Time course of butanol, isopropanol production and glucose consumption in the fed-batch fermentation with in situ extraction of biodiesel (ratio of 1:1); **b** time course of growth and pH in the batch fermentation with in situ extraction of biodiesel (ratio of 1:1)

It is well known that lipophilic butanol leads to low butanol titer due to the toxicity to cells, which in turn contributes to the high cost of product recovery. In situ removal of toxic solvent could not only minimize butanol inhibition, but also improve the final butanol titer [1, 2]. Biodiesel has been proved as an ideal extractant for butanol production without toxicity to the clostridial cells, which has a high partition coefficient for butanol (1.04), isopropanol (1.08), and low partition coefficients for acetone (0.18). To further improve the final isopropanol–butanol titer from glucose by strain NJP7, a fed-batch fermentation using biodiesel as in situ extractant with a volume ratio of 1:1 (fermentation medium:biodiesel = 1:1; added at the beginning) was carried out accordingly. As shown in Fig. 4a, 60 g/L of initial glucose could be rapidly utilized after 72 h of fermentation, and 16.43 (solvent phase: 8.41 g/L vs medium phase: 8.02 g/L) and 3.26 g/L (solvent phase:1.75 g/L vs medium phase: 1.51 g/L) of total butanol and isopropanol occurred, separately, which corresponds to 34.56 and 69.79% higher than those obtained in the control (butanol: 12.21 g/L; isopropanol: 2.21 g/L) (Table 2). When another 30 g/L of glucose was dosed into the fermentation medium, butanol and isopropanol production could be further improved to 25.58 and 5.24 g/L, respectively, with 14.35 g/L of glucose leftover after 144 h of fermentation (Fig. 4a). 13.21 and 12.37 g/L of butanol occurred in the biodiesel and fermentation broth phases; meanwhile, 2.78 and 2.46 g/L of isopropanol were detected in the biodiesel and fermentation broth phases, respectively (data now shown). Meanwhile, the simultaneous extraction of solvents could lead to higher amount of biomass (OD = 6.65 for 1:1; OD = 5.56 for the control), which may also contribute to the high butanol production (Fig. 4b). During the late solventogenic phase, 5.21 g/L of acetone and small amount of acids (butyrate: 0.45 g/L vs acetate: 0.36 g/L) were detected in the fermentation broth. Biodiesel has a partition coefficient of 1.2 for butyric acid, 1.1 for acetic acid, and 0.18 for acetone, so only partial acids (0.18 g/L for butyric acid; 0.11 g/L for acetic acid) and acetone (0.97 g/L) were transmitted from the fermentation medium to the solvent phase, which also resulted in higher pH values (above 5.5) during the fermentation with addition of biodiesel (Fig. 4b). So the butanol- and isopropanol-enriched biodiesel with negligible acetone and VFAs could be directly applied to the biofuel sector, which would further eliminate the subsequent extractant recycling.

### Direct butanol production from hemicelluloses in CBP by *Clostridium* sp. strain NJP7

**Fig. 5:**
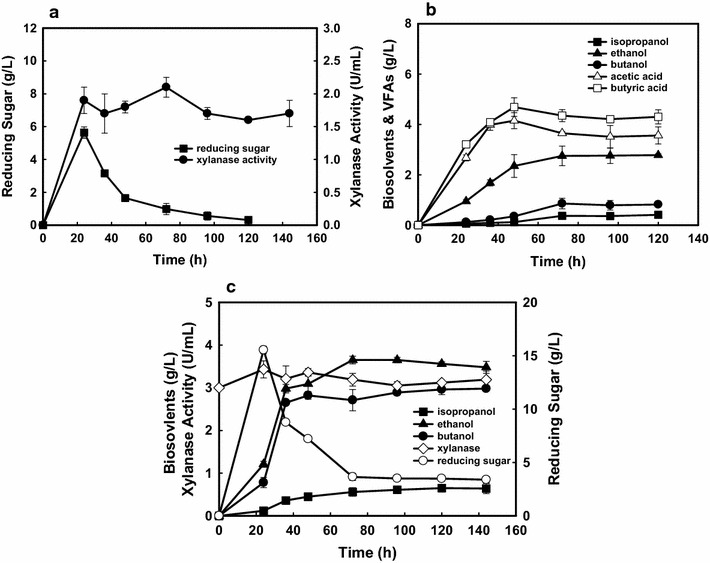
Metabolic profiles (**a**) and enzymatic activities (**b**) by *Clostridium* sp. strain NJP7 when amended with 60 g/L of birchwood xylan at 35 °C. Profiles of reducing sugars and metabolic profiles with addition of commercial xylanase and 60 g/L of birchwood xylan by *Clostridium* sp. strain NJP7 (**c**)

As discussed previously, the desired breakdown of polymers like xylan can be achieved through an enzymatic system collectively called xylanases [13]. Genomic sequence analysis of solventogenic *C. beijerinckii* and *C. acetobutylicum* has shown the presence of several xylanase related genes; however, there are only few studies about direct butanol production from hemicelluloses due to the low expression levels of xylanases [18, 19]. Therefore, direct fermentation strategies for butanol production were subsequently investigated using *Clostridium* sp. strain NJP7 in the presence of 60 g/L xylan (Fig. 5). As shown in Fig. 5a, strain NJP7 was capable of efficiently secreting xylanase (1.75 U/mL) and leading up to 5.62 g/L of accumulated reducing sugars in the medium during the first 24 h. Meanwhile, strain NJP7 could simultaneously convert these reducing sugars to biosolvents, such as 0.21 g/L of isopropanol, 0.83 g/L of butanol, and 2.79 g/L of ethanol (Fig. 5b). Hence, it can be concluded that culture NJP7 possesses both xylanolytic and solventogenic properties when cultivated in xylan-amended medium.

It also should be noticed that higher amounts of ethanol and VFAs (2.40 g/L acetate and 4.21 g/L butyrate) were detected when using xylan as the sole carbon source, which is contrary to our finding that only acetone–isopropanol–butanol and lower amount of VFAs were detected in monosaccharide (glucose)-amended medium (Fig. 2). It is known that cellulose/hemicellulose degradation is one of the rate-limiting steps for butanol production in CBP. The lower butanol and higher VFA production could be due to the low level of xylan hydrolysis, which leads to insufficient formation of the reducing sugars necessary for butanol production [20, 21]. Also in the view of reducing factors, two more NADH are needed for butanol formation by reassimilation of butyric acid than ethanol, which would facilitate butanol rather than ethanol production (Fig. 3) [2]. Indeed, when 3 U/mL of commercial xylanases was supplemented into the medium initially, higher accumulated reducing sugars (15.54 vs 5.62 g/L) at time of 24 h could lead to 159% of butanol increase (2.98 vs 0.83 g/L); however, ethanol only shows slight increase of 25% (3.48 vs 2.79 g/L) (Fig. 5c). Meanwhile, the slow reducing sugar feeding rate when using hemicelluloses would enable to efficiently convert acetone to isopropanol with no acetone formation. These findings are different from previous studies, where they show that acetone gene was suppressed when using polysaccharides, such as cellulose for solvent production [22].

### Improvement of butanol production from hemicelluloses via temperature- and pH-shifted strategy

The optimal pH for butanol production from monosaccharide (glucose) varied from 5.5 to 6.0 (Fig. 2); however, culture NJP7 showed low cell growth under such pH arranges when xylan was used as the sole carbon source, whose growth only occurred at pH above 6.0, indicating that there is a different pH requirement for xylanase activity and butanol formation. To better elaborate the relationship, characterization of the xylanase from strain NJP7 was carried out accordingly.

**Fig. 6:**
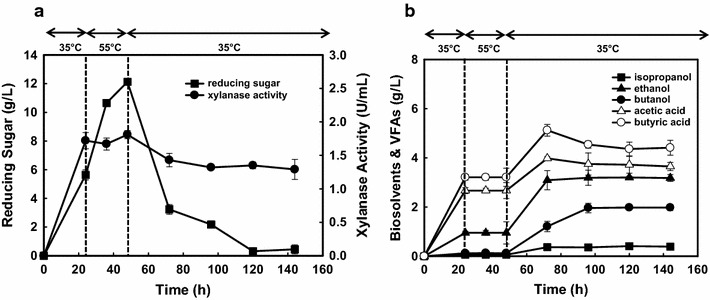
Metabolic profiles (**a**) and enzymatic activities (**b**) by *Clostridium* sp. strain NJP7 when amended with 60 g/L of birchwood xylan using temperature-shift strategy. *Arrow* means the inoculation point of *Clostridium* sp. strain NJP7 at 35 °C

During industrial application, whole cells are usually used as the source of enzymes, but the efficiency can be improved using purified enzymes or by excluding certain other unwanted enzymes [13]. Hence, the crude xylanase from culture NJP7 was firstly partially purified by ammonium sulfate fractionation (30–70% saturation), which gave 2.2-fold with a yield of 65.3%. This partially purified xylanase showed the most stable activity at pH 6.5, differing from the pH value required for butanol production (5.5–6.0). The enzyme activity beyond this range dropped dramatically, such as at pHs below 5.0 and above 8.0. The optimum temperature for xylanolytic hydrolysis reactions was 55 °C and thermostability studies showed that xylanase from strain NJP7 could retain 90% activity at 55 °C after 1 h of incubation. Based on these findings, a temperature- and pH-shifted strategy was designed. Specifically, strain NJP7 was cultivated at 35 °C and pH of 6.5 first for 24 h, and 1.7 U/mL of extracellular xylanase was released into the culture medium (Fig. 6a). Then the temperature was shifted to the optimum condition of 55 °C for efficient xylan hydrolysis. As shown in Fig. 5a, this will allow more efficient hydrolysis of xylan and lead to higher accumulated reducing sugars concentration (12.15 vs 5.86 g/L) as compared to the control (Fig. 5a). After 24 h, temperature and pH will be reduced to 35 °C and 5.5 and new cultures of NJP7 were inoculated to the medium. The inoculation of new strain NJP7 led to the rapid utilization of the sugars accumulated in the medium and formation of additional fermentation products (0.54 g/L isopropanol, 3.24 g/L ethanol, 2.06 g/L butanol, 4.21 g/L butyric acid, and 3.79 g/L acetic acid) (Fig. 6b). Although different temperature requirements would increase the operational costs in this temperature-shift strategy where enzymes would perform their most optimal function of hydrolysis, the elimination for enzymes production and further purification, which are main contributors to the overall costs of producing biofuel from biomass, would make this process more economically competitive and feasible compared to the one in which hydrolytic enzymes are produced by other microbes, followed by enzymes concentration and purification. And especially, this strategy will also provide a platform for other biofuels or biochemicals production from lignocellulosic wastes.

Currently, many efforts have been focusing on achievement of direct biobutanol production from lignocellulose through CBP (Table 3). Among the previously reported metabolically engineered strains, *C. thermocellum* with introduction of *kivd*, *ilvBN*, *ilvC*, and *ilvD* was able to generate 5.4 g/L of isobutanol from cellulose, which could probably be considered as the best isobutanol producer from lignocellulose [24]. More recently, a wild-type solventogenic *C. pasteurianum* GL11 was reported to produce 1.48 g/L of butanol with elimination of acetone from xylan in CBP [26]. Compared to these studies, *Clostridium* sp. strain NJP7 also shows significant advantages in several aspects, including direct conversion of xylan into butanol via CBP and co-production of more value-added product, isopropanol. Moreover, 2.06 g/L of butanol, 3.24 g/L of ethanol, and 0.54 g/L of isopropanol were achieved with a total amount of 5.84 g/L solvents, representing the highest solvent production directly from lignocellulose. These metabolic properties within strain NJP7 can greatly improve the economic viability of biobutanol production both in terms of the associated substrate costs and elimination of acetone formation. Nevertheless, further studies were still needed to improve the solvent titer to achieve comparable levels when using starchy-based materials.

**Table 3:** Comparison of direct biobutanol production from cellulose and hemicellulose by different *Clostridium* sp. strains

Organism	Genotype	Substrate	Butanol (g/L)	Total solvents (g/L)	References
*C. cellulolyticum*	Genetically modified^a^	Crystalline cellulose	0.66	0.66	[23]
*C. thermocellum*	Genetically modified^b^	Crystalline cellulose	5.4	5.4	[24]
*C. cellulovorans*	Genetically modified^c^	Crystalline cellulose	1.42	3.02	[25]
*C. pasteurianum* GL11	Wild type	Birchwood xylan	1.48	6.60	[26]
*Clostridium* sp. strain NJP7	Wild type	Birchwood xylan	2.06	5.84	This study

^a^Strain with introduction of *kivd*, 2-ketoacid decarboxylase; *yqhD* alcohol dehydrogenase/aldehyde reductase, *alsS* acetolactate synthase, *ilvC* acetohydroxyacid isomeroreductase, *ilvD* dihydroxy-acid dehydrogenase

^b^Strain with introduction of *kivd*, 2-ketoacid decarboxylase, *ilvBN* acetohydroxy acid synthase (AHAS), *ilvC* acetohydroxyacid isomeroreductase, *ilvD* dihydroxy-acid dehydrogenase

^c^Strain with introduction of aldehyde/alcohol dehydrogenase (*adhE2*)

## Conclusion

The newly isolated wild-type *Clostridium* sp. strain NJP7 synthesizes butanol through a fermentative acetone–isopropanol–butanol pathway and shows direct butanol production from monosaccharide via CBP. The final butanol and isopropanol titers could reach 25.58 and 5.24 g/L, respectively, via manipulations of reducing cofactors and in situ extraction with biodiesel. More importantly, strain NJP7 shows direct butanol production from xylan and a final of 2.06 g/L butanol was achieved via a temperature-shifted strategy. These findings in this study thus offer fundamental knowledge for the future development of economically viable alternative fuel production strategies.

## Methods

### Isolation, culture growth, and fermentation experiments

During the experiments, microorganisms (originated from the grass decompose soil, Jiangsu Province, China) were cultivated in a reduced mineral salts medium. The procedure to prepare the medium is briefed as follows: the serum bottles were spiked with a medium containing 1 g/L of NaCl, 0.5 g/L of MgCl_2_·6H_2_O, 0.2 g/L of KH_2_PO_4_, 0.3 g/L of NH_4_Cl, 0.3 g/L of KCl, 0.015 g/L of CaCl_2_·H_2_O, 0.2 g/L of MgSO_7_·H_2_O, and being amended with 60 g/L of glucose or xylan. In addition, 1 mL of trace element solution, 1 mL of Na_2_SeO_3_–Na_2_WO_4_ solution, and 10 mg of resazurin were added per liter of medium. After the medium was boiled and cooled down to room temperature (20–25 °C) under N_2_, reductants Na_2_S, l-cysteine, and _DL_-dithiothreitol were added to a final concentration of 0.2, 0.2, and 0.5 mM, respectively [21]. Subsequently, NaHCO_3_ was added to the medium and the pH was adjusted to 7.0. Then, 50 mL liquid medium was dispensed into 160-mL bottles, which were sealed with butyl stoppers, autoclaved for 20 min, and cooled down to room temperature (20–25 °C). After five transfers in birchwood xylan-amended medium, the enrichment culture (0.1 mL; successively diluted to 10^−5^ times) was repeatedly streaking on agar plates poured with mineral salts medium containing birchwood xylan as the sole carbon source. After incubation for 6 days, Congo red staining was used to indicate xylanase activities of the colonies. The xylanase activity of each colony was determined by measuring the zone of clearance on the agar plates. Through such processes, a pure bacterial culture, designated strain NJP7, was obtained. All the enrichment, isolation, and cultivation were performed in an anaerobic chamber filled with mixed gas (90% N_2_, 5% CO_2_, and 5% H_2_) and operated at 25 °C.

## 16S rRNA gene sequencing and phylogenetic analysis of strain NJP7

The genomic DNA of cell pellets from isolate NJP7 was extracted and purified with DNeasy Tissue Kit (Qiagen GmbH, Germany) according to the manufacturer’s instructions with minor modifications. With the genomic DNA of strain NJP7 as a template, PCR amplification of the 16S rRNA genes was performed with a pair of universal bacterial primer 8F (5′-AGAGTTTGATCCTGGCTCAG-3′) and 1392R (5′-ACGGGCGGTGTGT-3′) [27]. After cleaning the PCR products with a PCR purification kit (Qiagen GmbH, Germany), the 16S rRNA genes were sequenced on a ABI DNA Sequencer. After Basic Local Alignment Search Tool (BLAST), the 16S rRNA gene sequences of the isolate and other closely related strains were aligned using CLUSTAL X software [28]. Phylogenetic trees were constructed using the neighbor-joining method [29] implemented in the MEGA program [30]. The topologies of the tree were evaluated by bootstrap analysis, based on 1000 replicates. The nucleotide sequence of culture NJP7 was deposited in the GenBank under an accession number of KX378860.

### Enzymatic assays

The activities of butanol dehydrogenase (BDH) were measured by monitoring NADH consumption at 365 nm according to the method described before [31] with some modifications. Cells were collected from 100 mL of fermentation broth by centrifugation at 10,000*g* for 10 min using tightly sealed centrifuge tubes purged by N_2_ gas. The cell pellet was washed with Tris–HCl buffer (0.1 M, pH 7.5) once and resuspended in 5 mL of the same Tris–HCl buffer. Lysis was carried out using a French press with one passage at 77 MPa. Supernatant was collected by centrifugation at 15,000*g* for 10 min and used for enzyme activity assay. Enzyme activity was calculated on the basis of a molar NADH extinction coefficient of 3.4 cm^−1^ mM^−1^. One unit of enzyme activity was defined as the amount of enzyme which converts 1 μmol NADH per minute under the reaction conditions. The secondary alcohol dehydrogenase activity (s-ADH) was measured anaerobically at 25 °C in the direction of acetone reduction (NADPH oxidation) by monitoring the decrease in A 340 as an indication of the disappearance of NADPH. The assay was performed as described elsewhere [32]. One unit is defined as the amount of enzyme that oxidized 1 μmol min^−1^ of NADPH under these conditions. The extinction coefficient of NADPH at 340 nm was 6.22 mM^−1^ cm^−1^. Protein concentration in cell extract was determined using the Bio-Rad protein assay kit with bovine serum albumin as standard.

Xylanase activity was measured according to Bailey et al. [33] when serial enzyme dilutions were amended with 1% (w/v) birchwood xylan and 0.05 M glycine/NaOH buffer (pH 8.0). This mixture was incubated at 50 °C for 10 min. Reducing sugars released from xylanase were measured using the 3,5-dinitrosalicylic acid (DNS) method [34]. One international unit (U) of each enzyme was defined as the enzymatic activity required for the release of 1 µmol xylose equivalents per unit volume and minute of reaction.

The pH stability of xylanase was assessed at pH ranging from 4.0 to 12.0 at 60 °C after 30 min incubation. To determine the thermostability of xylanase activity, the xylanase was incubated at different temperatures (60–80 °C) in the absence of substrate. After incubating for 1 h, the residual xylanase activity was determined at 70 °C for 10 min [33].

### Analytic methods

Fermentation broth samples were analyzed for biomass growth, acetone, isopropanol, ethanol, and butanol concentration. Reducing sugars were estimated by the dinitrosalicylic acid (DNS) method with xylose as the standard [34]. Biomass was determined by measuring turbidity at 600 nm with appropriate dilution using a UV–visible spectrophotometer (Lambda-25, Perkin-Elmer, USA). Similarly, culture broths were centrifuged immediately after sampling at 10,000*g* for 10 min at 4 °C and the supernatant fluids were then stored at −20 °C for analysis. Glucose was analyzed by a 1200 Series HPLC system (Agilent Technologies Inc.) equipped with an Aminex HPX-87H column (Bio-Rad, Richmond, CA, USA) and a Refractive Index Detector (RID). The samples were run at 75 °C with 0.6 mL/min eluent of 5 mM sulfuric acid. Biosolvents (i.e., acetone, isopropanol, ethanol, and butanol) were measured by a gas chromatography (GC, model 7890A; Agilent Technologies, USA) on a Durabond (DB)-WAXetr column (30 m × 0.25 mm × 0.25 µm; J&W, USA) equipped with a flame ionization detector (FID). The oven temperature was initially held at 60 °C for 2 min, increased at 15 °C/min to 230 °C, and held for 1.7 min. Helium was used as the carrier gas, with a column flow of 1.5 mL/min. Five-point standard curves were obtained by running standard solutions containing acetone, isopropanol, butanol, and ethanol.

## Abbreviations

CBP: consolidated bioprocessing
ABE: acetone–butanol–ethanol
AIB: acetone–isopropanol–butanol
s-ADH: secondary alcohol dehydrogenase
BDH: butanol dehydrogenase

## References

[1] Lee SY, Park JH, Jang SH, Nielsen LK, Kim J, Jung KS (2008). Fermentative butanol production by Clostridia. Biotechnol Bioeng.

[2] Zhang YH (2015). Production of biofuels and biochemical by in vitro synthetic biosystems: opportunities and challenges. Biotechnol Adv.

[3] Gu Y, Jiang Y, Wu H, Liu X, Li Z, Li J, Xiao H, Shen Z, Dong H, Yang Y, Li Y, Jiang W, Yang S (2011). Economical challenges to microbial producers of butanol: feedstock, butanol ratio and titer. Biotechnol J.

[4] Lee J, Jang YS, Choi SJ, Im JA, Song H, Cho JH, do Seung Y, Papoutsakis ET, Bennett GN, Lee SY (2011). Metabolic engineering of *Clostridium acetobutylicum* ATCC 824 for isopropanol-butanol-ethanol fermentation. Appl Environ Microbiol.

[5] Papa AJ. Propanols in: Ullmann’s encyclopedia of industrial chemistry. Wiley-VCH, Weinheim. 2005; doi: 10.1002/14356007.a22_173.

[6] Hanai T, Atsumi S, Liao JC (2007). Engineered synthetic pathway for isopropanol production in *Escherichia coli*. Appl Environ Microbiol.

[7] George HA, Johnson JL, Moore WE, Holdeman LV, Chen JS. Acetone, isopropanol, and butanol production by *Clostridium beijerinckii.* (syn. *Clostridium butylicum*) and *Clostridium aurantibutyricum*. Appl Environ Microbiol. 1983;45:1160–63. 10.1128/aem.45.3.1160-1163.1983 PMC242427 16346237

[8] Survase SA, Jurgens G, van Heiningen A, Granstrom T (2011). Continuous production of isopropanol and butanol using *Clostridium beijerinckii* DSM 6423. Appl Microbiol Biotechnol.

[9] Shaheen R, Shirley M, Jones DT (2000). Comparative fermentation studies of industrial strains belonging to four species of solvent-producing clostridia. J Mol Microbiol Biotechnol.

[10] Ng ZR, Takahashi K (2013). Liu Z. Isolation, characterization and evaluation of hyper 2-propanol producing bacteria from Singapore environment. World J Microbiol Biotechnol.

[11] Qureshi N, Blaschek HP (2001). ABE production from corn: a recent economic evaluation. J Ind Microbiol Biotechnol.

[12] Zhang J, Wang M, Gao M, Fang X, Yano S, Qin S, Xia R (2013). Efficient acetone-butanol-ethanol production from corncob with a new pretreatment technology—wet disk milling. Bioenerg Res..

[13] Bajpai P (1997). Microbial xylanolytic enzyme system: properties and applications. Adv Appl Microbiol.

[14] Beg Q, Kapoor M, Mahajan L, Hoondal G (2001). Microbial xylanases and their industrial applications: a review. Appl Microbiol Biotechnol.

[15] Chen CK, Blaschek HP (1999). Effect of acetate on molecular and physiological aspects of *Clostridium beijerinckii* NCIMB 8052 solvent production and strain degeneration. Appl Environ Microbiol.

[16] Holt RA, Stephens GM, Morris JG (1984). Production of solvents by *Clostridium acetobutylicum* cultures maintained at neutral pH. Appl Environ Microbiol.

[17] Chen JS, Hiu SF (1986). Acetone-butanol-isopropanol production by *Clostridium beijerinckii* (synonym, *Clostridium butylicum*). Biotechnol Lett.

[18] Nölling J, Breton G, Omelchenko MV, Makarova KS, Zeng Q, Gibson R, Lee HM, Dubois J, Qiu D, Hitti J, Wolf YI, Tatusov RL, Sabathe F, Doucette-Stamm L, Soucaille P, Daly MJ, Bennett GN, Koonin EV, Smith DR (2001). Genome sequence and comparative analysis of the solvent-producing bacterium *Clostridium acetobutylicum*. J Bacteriol.

[19] Wang Y, Li X, Mao Y, Blaschek HP (2012). Genome-wide dynamic transcriptional profiling in *Clostridium beijerinckii* NCIMB 8052 using single-nucleotide resolution RNA-Seq. BMC Genom.

[20] Petitdemange E, Fond O, Caillet F, Petitdemange H, Gay R (1983). A novel one step process for cellulose fermentation using mesophilic cellulolytic and glycolytic *Clostridia*. Biotechnol Lett.

[21] Widdel F, Hansen TA. The dissimilatory sulfate-and sulfur-reducing bacteria. The prokaryotes: a handbook on the biology of bacteria: ecophysiology, isolation, identification, applications. 1992;vol. 1. (Ed. 2):582–624.

[22] Nakayama S, Kiyoshi K, Kadokura T, Nakazato A (2011). Butanol production from crystalline cellulose by cocultured *Clostridium thermocellum* and *Clostridium saccharoperbutylacetonicum* N1-4. Appl Environ Microbiol.

[23] Higashide W, Li Y, Yang Y, Liao JC (2011). Metabolic engineering of *Clostridium cellulolyticum* for production of isobutanol from cellulose. Appl Environ Microbiol.

[24] Lin PP, Mi L, Moriok AH, Yoshino MM, Konishi S, Xu SC, Papanek BA, Riley LA, Guss AM, Liao JC (2015). Consolidated bioprocessing of cellulose to isobutanol using *Clostridium thermocellum*. Metab Eng.

[25] Yang XR, Xu MM, Yang ST (2015). Metabolic and process engineering of *Clostridium cellulovorans* for biofuel production from cellulose. Metab Eng.

[26] Xin F, Wang C, Dong W, Zhang W, Wu H, Ma J, Jiang M (2016). Comprehensive investigations of biobutanol production by a non-acetone and 1,3-propanediol generating *Clostridium* strain from glycerol and polysaccharides. Biotechnol Biofuels.

[27] Maniatis T, Fritsch EF, Sambrook J (1982). Molecular cloning: a laboratory manual. Cold Spring Harbor Laboratory.

[28] Thompson JD, Gibson TJ, Plewniak F, Jeanmougin F, Higgins DG (1997). The CLUSTAL_X windows interface: flexible strategies for multiple sequence alignment aided by quality analysis tools. Nucleic Acids Res.

[29] Saitou N, Nei M (1987). The neighbor-joining method: a new method for reconstructing phylogenetic trees. Mol Biol Evol.

[30] Kumar S, Tamura K, Nei M (2004). MEGA3: integrated software for molecular evolutionary genetics analysis and sequence alignment. Briefi Bioinforms..

[31] Dürre P, Kuhn A, Gottward M, Gottschalk G (1987). Enzymatic investigations on butanol dehydrogenase and butyraldehyde dehydrogenase in extracts of *Clostridium acetobutylicum*. Appl Microbiol Biotechnol.

[32] Hiu SF, Zhu CX, Yan RT, Chen JS (1987). Butanol-ethanol dehydrogenase and butanol-ethanol- isopropanol dehydrogenase: different alcohol dehydrogenases in two strains of *Clostridium beijerinckii* (*Clostridium butylicum*). Appl Environ Microbiol.

[33] Bailey MJ, Biely P, Poutanen K (1992). Interlaboratory testing of methods for assay of xylanase activity. J Biotechnol.

[34] Miller GL (1959). Use of dinitrosalicylic acid reagent for determination of reducing sugar. Anal Chem.

